# Surveying Health-Related Knowledge, Attitudes, and Behaviors of U.S.-Based Residents Traveling Internationally to Visit Friends and Relatives

**DOI:** 10.4269/ajtmh.20-0508

**Published:** 2020-09-21

**Authors:** Erica Rapheal, Steven T. Stoddard, Kathryn B. Anderson

**Affiliations:** 1 University of Minnesota, Minneapolis, Minnesota;; 2 Emergent BioSolutions Inc., Gaithersburg, Maryland;; 3 Department of Microbiology and Immunology, State University of New York-Upstate Medical University, Syracuse, New York;; 4 Department of Medicine, State University of New York-Upstate Medical University, Syracuse, New York;; 5 Institute for Global Health and Translational Sciences, State University of New York-Upstate Medical University, Syracuse, New York

## Abstract

U.S. residents traveling internationally to regions with increased risk of infectious diseases infrequently seek pretravel health care. First- and second-generation immigrants traveling to their countries of origin and visiting friends and relatives (VFRs) have increased risk of certain infectious diseases and are more likely to participate in high-risk activities. In an online survey of 994 U.S. residents with two foreign-born parents who went on at least one international trip to an at-risk country (defined as having a typhoid vaccine recommendation) in the prior 3 years, respondents were questioned about their international travel over the previous 3 years and their knowledge and individual risk of disease. Participants reported infrequently seeking pretravel health information (32% of trips) or consulting a healthcare provider before their trips (15% of trips). Participants reported seeking pretravel health information less often for VFR trips home (22%) than to other regions (30%). Perceived risk of disease was directly associated with seeking pretravel health information (82% for the highest and 13% for the lowest perceived risk), consulting a healthcare provider (55% for the highest and 5% for the lowest perceived risk), and reporting travel-associated illness (54% for the highest and 10% for the lowest perceived risk). Respondents were generally knowledgeable about cholera, hepatitis B, malaria, and rabies but had low knowledge of hepatitis A and typhoid. Understanding where VFR travelers lack understanding of disease transmission and which travelers are ideal targets for interventions has the potential to shape physician recommendations and public health strategy in this vulnerable population.

## INTRODUCTION

More than 20% of people in the United States are first- or second-generation immigrants^1^; for these individuals, traveling back to their country of origin can present serious health risks. International travel for visiting friends and relatives (VFRs) is defined by the U.S. CDC as first- or second-generation immigrants traveling to a low- or lower-middle–income country to visit friends or relatives and having a home stay during their trip. Visiting friend and relative travel has been associated with increased risk of travel-related illness including malaria, viral hepatitis, and HIV/AIDS.^2^ Visiting friend and relative travelers typically have longer trip duration and more opportunities for disease exposure, and are more likely to report risky behavior, such as brushing teeth with tap water or eating uncooked food.^3^

Among U.S. residents traveling internationally, only approximately 12% visit a healthcare provider for pretravel care.^4^ Visiting friend and relative travelers may be even less likely to seek healthcare advice before traveling because of cost, language barriers, presumption of immunity, or lack of awareness of travel medicine as a clinical specialty in their country of residence.^5^ Visiting friend and relative travelers from the United States more commonly visit urban destinations and are more likely to be children than non–VFR travelers.^6^ Visiting friend and relative travelers also often travel despite being pregnant or having multiple medical problems, which can increase susceptibility to infectious and noninfectious illnesses.^5^ When they do seek pretravel health care and counseling, VFR travelers more frequently present for care within the three weeks before departure, complicating the administration of recommended multidose vaccines.^7^ In addition, VFR travelers are less likely to receive prescription antibiotics for traveler’s diarrhea, more likely to decline vaccines,^6,8^ and infrequently use measures such as bed netting to prevent mosquito bites in at-risk areas.^9^ Staying with family may also reduce dietary autonomy for VFR travelers, increasing the likelihood of drinking untreated water and eating uncooked foods.^10^

Understanding the attitudes and behaviors of VFR travelers regarding pretravel health care, and identifying barriers to accessing it continue to be important for developing effective strategies and interventions to improve care.^11^ Because VFR travelers may receive information from multiple sources (Internet, travel clinic, and primary care physician),^12–15^ it is important that these interventions do not focus only on travel physicians. The potential for intervention in at-risk groups is high, given that VFR travelers commonly travel multiple times to their country of origin.^16^

Using a post-travel survey, we evaluated factors associated with pretravel health-seeking behaviors and risk perceptions among a cohort of individuals who recently participated in VFR travel. Risk factors associated with VFR travel are regularly captured in large datasets such as GeoSentinel and TravEpiNet.^6,8,16–18^ However, our dataset is unique because, unlike previous studies, it is not limited to individuals who sought pre- or post-travel care at a travel clinic and does not focus on individuals traveling to one destination. This broadens the scope of the study and reduces the potential for bias associated with the care-seeking behavior. This study also collects information about how members of this cohort behaved on non-VFR trips, providing insight into how the act of traveling to visit friends or relatives impacts decisions and outcomes independent of demographic factors associated with this type of traveler. The aim of this study was to identify aspects of VFR travel associated with increased risk of exposure and illness as well as opportunities for prevention through counseling and vaccination.

## METHODS

### Visiting friend and relative traveler survey.

Participants were recruited through the commercial market research agency Survey Sampling International (www.surveysampling.com), which maintains a database of more than 7 million voluntary market research participants throughout the United States (full survey text in Supplemental Appendix A). Invitations were sent electronically, and 8,961 individuals completed the survey. Respondents were U.S. residents with two foreign-born parents who had completed at least one international trip in the previous 3 years. Individuals of Mexican origin, those younger than 26 years, those who did not travel to visit friends or relatives in the previous 3 years, and those who did not travel to an at-risk country (defined as a country with a typhoid vaccine recommendation as per the CDC and International Association for Medical Assistance to Travellers)^19,20^ were excluded from this group for a total participant population of *n* = 994 (Supplemental Appendix B). For the purposes of this study, we defined a VFR traveler as any individual who is an immigrant to the United States or whose parents were immigrants, and who has traveled to their or their parents’ country of origin to visit friends or relatives in the last 3 years. This defines VFR travelers as a cohort rather than a type of travel. We use the term “VFR trip home” to denote a VFR traveler who is traveling to their or their parents’ country of origin on a given trip. Individuals younger than 26 years were excluded to better capture individuals making their own travel decisions, rather than those traveling with a parent.

Survey participation was voluntary. Survey questions were related to participant demographics (age, gender, ethnicity, and language), income and education level, healthcare utilization (medical insurance, frequency of access, and vaccines), and disease awareness (knowledge about select diseases and risk). Respondents were asked specific questions about their international travels over the previous 3 years. For up to three of the participants’ most recent international trips, participants were asked questions regarding travel planning, travel arrangements, destinations, health concerns, pretravel health consultations, and administration/acceptance of travel vaccines. Each respondent provided their demographic information and knowledge of disease one time, with the option to provide responses for between 1 and 3 trips. This accounted for individuals who behaved differently or traveled for different reasons on separate trips. Individuals with more than three international trips in the previous 3 years were asked to report only the three most recent trips. The survey required participants to provide only one answer per question for most questions (i.e., participants were not allowed to list two reasons for traveling on a given trip).

Travelers were asked to self-report their knowledge about given diseases and how they believe those diseases are transmitted (person-to-person, airborne, waterborne, animal contact, etc.). Responses to questions regarding transmission were categorized as correct or incorrect. Travelers were asked what they believed their risk of disease was when traveling on a scale of 1 (low) to 5 (high). For analysis, risk was subset into high (3–5) and low (1–2). The actual risk of transmission was approximated using disease risk maps from the WHO^21–28^ based on the participant’s first reported region of travel.

### Statistical analyses.

All analyses were performed using SAS 9 (SAS Institute Inc., Cary, NC). The chi-square test was used to determine significance of demographic variables (age, gender, education level, immigrant status, or health insurance type) in relation to any type of pretravel health-seeking behavior, pretravel consultation with a healthcare provider, and reported post-travel illness. Demographic variables were assessed per traveler rather than per trip, which precluded the need to account for travelers who took multiple trips in chi-square analysis.

Participants who sought out pretravel health information were those who selected “seek out travel health information” from a drop-down menu when asked how they had prepared for a given trip. Participants who consulted a healthcare practitioner before travel were those who reported seeking out travel health information and selected “spoke to or visited my healthcare practitioner” from a drop-down menu before a given trip. Participants were considered positive for a post-travel illness if they answered yes when asked whether they or a companion “got sick while traveling or shortly after returning to the United States.”

Only one trip was required to be to a country at risk for typhoid for that individual to be included in the study population; because participants could report up to three trips, trips to non–typhoid-endemic countries were reported by some participants and included in analysis of trip-level data. However, participants traveling to North America, Europe, Australia/New Zealand, Central Asia, and West Asia were few in number. Therefore, to maintain statistical power, North America, Europe, and Australia/New Zealand were grouped into other Western countries, and East Asia, Central Asia, and Western Asia were grouped into Eastern Asia.

Logistic regression analyses were used to identify variables associated with pretravel health information seeking and post-travel illness. Examination of significant risk factors in previous studies, as well as best-fit akaike information criterion (AIC) analysis, was used to determine which variables were included in the logistic model. To account for duplicate data from individuals with multiple trips, we used a mixed effects logistic regression model with random intercept. The following variables were condensed to streamline the model: 1) traveled with other adults included traveling with non-related adults, other family, or spouse; and 2) Asia included Southeast, South, and East Asia. Models were subset into VFR returning home travelers versus all trips.

## RESULTS

### Survey responses.

Of 8,961 respondents, a total of 1,002 met the inclusion criteria. Of these, 994 completed the survey (Supplemental Appendix B). Among those who completed the survey, 86% were foreign born and 14% were U.S. citizens with foreign-born parents. Participants reported a total of 2,228 trips in the prior 3 years, averaging 2.22 international trips per respondent.

#### Pretravel health behaviors.

Participants reported infrequently seeking pretravel health information (32% of travelers) or consulting a healthcare provider before their trips (15% of travelers). Demographic characteristics that were significant predictors of pretravel healthcare seeking were age (*P* = 0.017), education level (*P* = 0.0014), immigrant status (*P* = 0.0036), and type of insurance (*P* = 0.025) (Table 1). People older than 50 years, those with high school education or below, immigrants, and those without health insurance were less likely to report any pretravel healthcare seeking. Individuals who reported seeking any type of pretravel care were more likely to also report that someone on the trip got sick (*P* < 0.0001): 26% of those who sought care reported an illness, compared with 15% of those who did not seek care.

**Table 1:** Pretravel health behavior and outcomes based on demographics

	Sought pretravel health information before any trip			Consulted with healthcare provider before any trip			Travel-associated illness on any trip		
	No (*n* = 659), *n* (%)	Yes (*n* = 311), *n* (%)	*P*-value	No (*n* = 824), *n* (%)	Yes (*n* = 146), *n* (%)	*P*-value	No (*n* = 726), *n* (%)	Yes (*n* = 244), *n* (%)	*P*-value
Age-group (years)			0.017			0.2474			0.0018
26–50	475 (66)	250 (34)		610 (84)	115 (16)		524 (72)	201 (28)	
51–65	127 (77)	39 (23)		148 (89)	18 (11)		142 (86)	24 (14)	
65+	57 (72)	22 (28)		66 (84)	13 (16)		60 (76)	19 (24)	
Gender*			0.73			0.4352			0.8305
Female	341 (68)	162 (32)		425 (84)	78 (16)		384 (76)	119 (24)	
Male	224 (69)	101 (31)		281 (86)	44 (14)		246 (76)	79 (24)	
Education level			0.0014			0.1982			0.0582
Advanced Intl.	93 (67)	46 (33)		115 (83)	24 (17)		103 (74)	36 (26)	
Advanced U.S.	162 (65)	88 (35)		207 (83)	43 (17)		172 (69)	78 (31)	
College Intl.	143 (73)	54 (27)		173 (88)	24 (12)		153 (78)	44 (22)	
College U.S.	176 (62)	107 (38)		236 (83)	47 (17)		213 (75)	70 (25)	
≤ High school Intl.	48 (86)	8 (14)		52 (93)	4 (7)		46 (82)	10 (18)	
≤ High school U.S.	37 (82)	8 (18)		41 (91)	4 (9)		39 (87)	6 (13)	
Immigrant status			0.0036			0.1172			0.1218
Immigrant	587 (70)	256 (30)		722 (86)	121 (14)		638 (76)	205 (24)	
Nonimmigrant	72 (57)	55 (43)		102 (80)	25 (20)		88 (69)	39 (31)	
Health insurance type			0.025			0.0181			0.3064
Commercial/EMP	403 (66)	208 (34)		509 (83)	102 (17)		449 (73)	162 (27)	
Commercial/other	79 (71)	32 (29)		97 (87)	14 (13)		91 (82)	20 (18)	
Medicare/government	127 (67)	62 (33)		460 (94)	29 (6)		142 (75)	47 (25)	
No insurance	50 (85)	9 (15)		58 (98)	1 (2)		44 (75)	15 (25)	

EMP = employer; Intl. = international.

* Gender contains 142 missing values.

The only significant predictor of consulting a healthcare provider before travel was insurance status, with only 2% of persons without insurance consulting a healthcare provider before traveling. Age was the only significant predictor of reported post-travel illness (*P* = 0.0018), with those aged 26–50 or > 65 years more commonly reporting they or a companion got sick on any trip or soon after returning home.

Region of travel was significantly associated with all three outcomes. Individuals traveling to Africa were much more likely to report seeking pretravel health information, more likely to consult a healthcare provider, and more likely to report illness (Table 2). By contrast, those going to Latin America were less likely to report seeking travel-related health information or consulting a healthcare provider before traveling. Participants were less likely to report seeking pretravel health information for VFR trips home compared with other trips (22% versus 30%), but more likely to consult a healthcare provider if they did so (17% versus 12%). Perceived risk of disease was directly associated with any pretravel healthcare-seeking behavior (82% for the highest and 13% for the lowest perceived risk), consulting a healthcare provider (55% for the highest and 5% for the lowest perceived risk), and travel-associated illness (54% for the highest and 10% for the lowest perceived risk). Respondents who spent 3 months or more planning were more likely to seek care from a healthcare provider before travel (16% versus 10% of those who prepared for 0–2 months). Visiting friend and relative going home travelers were less likely to report illness in their party during travel than those going on other trips (16% versus 21%). Persons traveling with another individual were more likely to report illness during travel than those traveling alone.

**Table 2:** Pretravel health behaviors and outcomes for individual trips

	Sought pretravel health information			Consulted with healthcare provider before travel			Trip-associated illness		
	No (*n* = 1,644), *n* (%)	Yes (*n* = 545), *n* (%)	*P*-value	No (*n* = 1,868), *n* (%)	Yes (*n* = 301), *n* (%)	*P*-value	No (*n* = 1,794), *n* (%)	Yes (*n* = 375), *n* (%)	*P*-value
Region of travel			< 0.0001			< 0.0001			0.0465
Africa	38 (48)	41 (52)		48 (61)	31 (39)		58 (73)	21 (27)	
Eastern Asia	357 (78)	102 (22)		401 (87)	58 (13)		391 (85)	68 (15)	
Latin America and the Caribbean	525 (82)	113 (18)		591 (93)	47 (7)		536 (84)	102 (16)	
South Asia	188 (75)	63 (25)		314 (79)	86 (22)		196 (78)	55 (22)	
Southeast Asia	275 (69)	125 (31)		301 (88)	41 (12)		331 (83)	69 (17)	
Other Western countries	261 (76)	81 (24)		213 (85)	38 (15)		282 (82)	60 (18)	
Visiting friend and relative trip home			< 0.0001			0.0023			0.0084
Yes	1,190 (78)	327 (22)		539 (83)	113 (17)		1,276 (84)	241 (16)	
No	454 (70)	198 (30)		1,329 (88)	188 (12)		518 (79)	134 (21)	
Purpose of travel			< 0.0001			0.0523			0.359
Tourism	530 (70)	228 (30)		640 (84)	118 (16)		615 (81)	143 (19)	
Visit only	1,060 (80)	269 (20)		1,162 (87)	167 (13)		1,111 (84)	218 (16)	
Work/study	54 (66)	28 (34)		66 (80)	16 (20)		68 (83)	14 (17)	
Perceived risk (1 = low and 5 = high)			< 0.0001			< 0.0001			< 0.0001
1	997 (87)	147 (13)		1,063 (95)	61 (5)		1,026 (90)	118 (10)	
2	336 (71)	136 (29)		396 (84)	76 (16)		394 (83)	78 (17)	
3	230 (61)	144 (39)		282 (75)	92 (25)		276 (74)	98 (26)	
4	75 (51)	71 (49)		92 (63)	54 (37)		83 (57)	63 (43)	
5	6 (18)	27 (82)		15 (45)	18 (54)		15 (45)	18 (55)	
Travel companions			< 0.0001			< 0.0001			< 0.0001
None	556 (87)	86 (13)		598 (93)	44 (7)		597 (93)	45 (7)	
Children	446 (69)	204 (31)		526 (81)	124 (19)		495 (76)	155 (24)	
Non-related adults	51 (65)	28 (35)		64 (81)	15 (19)		61 (77)	18 (23)	
Other family	184 (74)	66 (26)		211 (84)	39 (16)		199 (80)	51 (20)	
Spouse	407 (74)	141 (26)		469 (86)	79 (14)		442 (81)	106 (19)	
Time spent preparing for trip (months)			< 0.0001			< 0.0001			0.517
0–2	658 (82)	149 (18)		729 (90)	78 (10)		673 (83)	134 (17)	
3+	986 (72)	376 (28)		1,139 (84)	223 (16)		1,121 (82)	241 (18)	

#### Self-reported knowledge.

In general, respondents were knowledgeable about cholera, hepatitis B, malaria, and rabies, but had low knowledge of hepatitis A and typhoid (Table 3). Perceived knowledge was mostly associated positively with actual knowledge.

**Table 3:** Self-reported knowledge of disease

Demonstrated knowledge of transmission	Self-reported knowledge of disease*			*P*-value
	High, *n* (%)	Low, *n* (%)	Total, *n* (%)	
Cholera				0.015
Right	571 (80)	129 (72)	700 (79)	
Wrong	139 (20)	50 (28)	189 (21)	
Hepatitis A				0.0019
Right	262 (32)	22 (18)	284 (30)	
Wrong	560 (68)	100 (82)	660 (70)	
Hepatitis B				0.80
Right	627 (74)	89 (75)	716 (74)	
Wrong	224 (26)	30 (25)	254 (26)	
Malaria				0.0037
Right	522 (79)	47 (64)	569 (77)	
Wrong	143 (22)	27 (36)	170 (23)	
Tuberculosis				0.024
Right	378 (58)	37 (45)	415 (56)	
Wrong	278 (42)	46 (55)	324 (44)	
Typhoid				0.0002
Right	348 (45)	52 (30)	400 (42)	
Wrong	422 (55)	122 (70)	544 (58)	
Rabies				0.0088
Right	681 (83)	84 (72)	765 (81)	
Wrong	144 (17)	32 (28)	176 (19)	
Japanese encephalitis				0.0007
Right	97 (47)	51 (30)	148 (39)	
Wrong	109 (53)	119 (70)	228 (61)	

* High = 3, 4, or 5; low = 1 or 2.

#### Predictors of pretravel care and post-travel illness.

For VFR trips home, significant logistic regression variables independently associated with seeking pretravel health care in a multivariate model were region of travel (*P* < 0.0001), travel companions (*P* < 0.0001), and time spent trip planning (*P* = 0.028) (Table 4). For all trips, variables significantly associated with seeking pretravel health care were type of travel (VFR going home or other) (*P* = 0.001), region of travel (*P* ≤ 0.0001), travel companions (*P* ≤ 0.0001), and time spent trip planning (*P* = 0.013). Variables not significantly associated with seeking pretravel health care were age, gender, and purpose of travel.

**Table 4:** Logistic regression model of any pretravel care seeking for individual trips

Variable	Estimate	95% CI		Pr > |Z|	Pr > ChiSq*
VFR trip home					
Intercept	−3.2	−4.0	−2.5	< 0.0001	
Age-group (years)					
26–50	0.30	−0.32	0.92	0.34	
65+	0.017	−0.71	0.74	0.96	
51–65	REF				0.38
Gender					
Female	0.10	−0.25	0.45	0.57	
Male	REF				0.57
Purpose of travel					
Tourism	0.30	−0.018	0.61	0.06	
Work/study	0.51	−0.097	1.1	0.10	
Visit only	REF				0.073
Region of travel					
Africa	1.7	0.87	2.6	< 0.0001	
Asia	0.86	0.45	1.3	< 0.0001	
Latin American and the Caribbean	REF				< 0.0001
Travel companions					
Other adults	0.79	0.41	1.2	< 0.0001	
Children	1.0	0.60	1.4	< 0.0001	
None	REF				< 0.0001
Time spent planning trip (months)					
3+	0.32	0.036	0.61	0.03	
0–2	REF				0.028
All trips					
Intercept	−3.0	−3.7	−2.3	< 0.0001	
VFR trip home					
No	0.47	0.18	0.76	0.00	
Yes	REF				0.001
Age-group (years)					
26–50	0.40	−0.019	0.81	0.06	
65+	0.01	−0.62	0.65	0.97	
51–65	REF				0.093
Gender					
Female	0.15	−0.15	0.45	0.32	
Male	REF				0.32
Purpose of travel					
Tourism	0.074	−0.18	0.33	0.56	
Work/study	0.61	0.04	1.2	0.04	
Visit only	REF				0.11
Region of travel					
Africa	1.3	0.52	2.02	0.00	
Asia	0.76	0.29	1.23	0.00	
Latin America and the Caribbean	0.16	−0.35	0.67	0.53	
Other Western countries	REF				< 0.0001
Travel companions					
Other adults	0.59	0.29	0.89	0.00	
Children	0.77	0.44	1.10	< 0.0001	
None	REF				< 0.0001
Time spent planning trip (months)					
3+	0.31	0.065	0.55	0.01	
0–2	REF				0.013

VFR = visiting friend and relative.

* Pr > ChiSq represents overall association for a given variable.

In a multivariate logistic regression analysis limited to VFR trips home, travel companions (*P* ≤ 0.0001) and purpose of travel (*P* = 0.024) were significant independent predictors of reported post-travel illness (Table 5). Variables not significantly associated with travel-associated illness for trips home were age, gender, region of travel, and whether pretravel care was sought. In a logistic regression analysis of all trips, significant independent associations were age (*P* = 0.016), travel companions (*P* < 0.0001), and whether pretravel health information was sought (*P* = 0.025). Variables not significantly associated with travel-associated illness for all trips were trip type, gender, and purpose of travel.

**Table 5:** Logistic regression model of reported post-travel illness for individual trips

Variable	Estimate	95% CI		Pr > |Z|	Pr > ChiSq*
VFR trip home					
Intercept	−3.8	−4.9	−2.7	< 0.0001	
Age-group (years)					
26–50	0.63	0.041	1.2	0.036	
65+	0.36	0.48	1.2	0.40	
51–65	REF				0.092
Gender					
Male	0.013	−0.37	0.39	0.95	
Female	REF				0.95
Region of travel					
Africa	1.17	0.29	2.1	0.010	
Asia	0.95	0.056	1.8	0.037	
Latin American and the Caribbean	REF				0.78
Travel companions					
Other adults	0.30	−0.82	1.41	0.60	
Children	0.13	−0.29	0.54	0.56	
None	REF				< 0.0001
Purpose of travel					
Tourism	0.86	0.38	1.3	0.000	
Visit only	1.1	6.0	1.6	< 0.0001	
Work/study	REF				0.024
Sought pretravel care					
Yes	0.345	−0.047	0.73	0.084	
No	REF				0.084
All trips					
Intercept	−4.03	−4.93	−3.12	< 0.0001	
VFR trip home					
No	1.08	−0.27	2.43	0.12	
Yes	REF				0.11
Age-group (years)					
26–50	0.73	0.23	1.2	0.004	
65+	0.55	−0.16	1.3	0.13	
51–65	REF				0.016
Gender					
Female	0.006	−0.32	0.33	0.97	
Male	REF				0.97
Purpose of travel					
Tourism	1.1	0.36	1.8	0.004	
Work/study	0.86	0.12	1.6	0.023	
Visit only	REF				0.49
Travel companions					
Other adults	0.83	0.43	1.2	< 0.0001	
Children	1.125	0.71	1.5	< 0.0001	
None	REF				< 0.0001
Sought pretravel care					
Yes	0.31	0.065	0.55	0.01	
No	REF				0.013
Purpose of trip home					
Other trips tourism*	−1.3	−2.63	0.11	0.072	
Other trips visit only*	−0.78	−2.21	0.65	0.28	
Other trips work/study*	REF				
VFR trip home tourism*	REF				
VFR trip home visit only*	REF				
VFR trip home work/study*	REF				0.054

VFR = visiting friend and relative.

* Pr > ChiSq represents overall association for a given variable.

## DISCUSSION

In our survey of more than 900 first- and second-generation U.S. citizens and immigrants traveling to countries with novel risk of infectious disease, participants reported low levels of any type of pretravel health-seeking behavior (32%), and even fewer individuals consulted a healthcare provider directly before their trips (15%). Participants reported seeking pretravel health information less often for VFR trips home (22%) than to other regions (30%). In our analysis, participants reported lower frequency of travel-associated illness after visiting their home countries versus other countries. Overall, older individuals, those with less education, immigrants, those without health insurance, and VFR travelers going home were less likely to report seeking any pretravel health information. Older people, in particular, are more likely to have comorbidities such as diabetes, renal disease, and heart disease, and therefore are at increased risk of complications from travel-related illnesses. For VFR trips home, variables significantly associated with seeking health information before traveling were travel region, travel companions, and time spent planning.

The low level of pretravel health behaviors is consistent with previous reports. In a Swiss survey of VFR travelers, 20% reported seeking care before traveling to at-risk countries.^2^ Among VFR travelers in the GeoSentinel Surveillance Network in 2006, 24% sought pretravel health advice before traveling.^17^ A 2007–2011 review of the same network reported that only 18% of VFR travelers who reported illness at a travel clinic upon return to their country of residence had sought advice before traveling.^18^ Another study in European travelers found that 31% of VFR travelers sought health advice before leaving.^29^ Collectively, these values are consistent with our findings for VFR travelers not taking a VFR trip home (30%), but higher than those for individuals taking VFR trips home (22%). These results are all lower than the overall rate of pretravel advice-seeking behavior among U.S. travelers to low- and lower-middle–income countries: about 54%.^30^

In our analysis, gender was not associated with seeking pretravel health information or consulting a healthcare provider. By contrast, in a study of tourists traveling to Peru, it was reported that women were significantly more likely to seek pretravel health information than men.^31^ Travel to Africa was not a significant factor in the likelihood of seeking pretravel health information or consulting a healthcare provider in this analysis. However, in a retrospective cohort study of international travelers, travel to Africa was associated with a higher likelihood of making a pretravel visit to a travel medicine clinic or primary care provider for consultation regarding traveler’s diarrhea.^32^

For VFR trips home, variables significantly associated with having a travel-associated illness were travel companions and purpose of travel. Among all trips in this analysis, reported illness was associated with the youngest and oldest age-groups, VFR travelers not going home, and traveling with children. It is generally accepted that VFR travelers are at greater risk of illness while traveling than non-VFR travelers.^33^ Given that our entire study population consisted of VFR travelers, we could not directly compare VFR and non-VFR travelers. Yet, the finding that VFR travelers who are traveling for non-VFR reasons more often reported sick was unexpected. It may be that such travelers maintain the perception that they are protected against health risks in places other than their home country. The estimated percentage of international travelers who have illness during travel varies widely in the literature, ranging from 6% to 87%,^34^ which makes meaningful comparison to our results difficult. In our analysis, among all trips, VFR trip status*travel was nearly statistically significant and therefore may be an effect modifier. Thus, it may have been beneficial to modify our definition of “VFR going home trip” to reflect that there may be a functional difference between VFR/non-VFR traveling for business or tourism and traveling for visiting only.

Participants’ perceived knowledge related to risks of infectious diseases generally corresponded with actual knowledge; however, for different diseases, there were very different levels of overall knowledge. In general, respondents were knowledgeable about transmission patterns of malaria, hepatitis B, cholera, and rabies but had low knowledge of typhoid and hepatitis A. Malaria is a serious cause of morbidity worldwide, with 219 million cases in 92 countries in 2017.^35^ Individuals who emigrate from malaria-endemic regions are very likely to have personal or secondhand experience with malaria infection. For hepatitis B, people may be generally knowledgeable about how the virus is spread but less aware of their risk of contracting it, given that only 11% of people who have it are aware of their infection.^36^ Participants had a more intermediate knowledge of transmission patterns of tuberculosis, which caused 1.5 million deaths in 2018 globally^37^; it may be that people are aware of the disease without fully knowing how it is spread.

Our results indicate that increased counseling on safe eating and drinking habits while traveling is needed for VFR travelers. The limited knowledge of typhoid is concerning, given that all participants who completed the survey reported traveling to a country with a typhoid vaccine recommendation (travel to a typhoid vaccination recommendation country was an inclusion criteria). Returning VFR travelers remain a significant source of typhoid infection in developing countries, despite low overall case counts.^3^ However, it has been previously reported that the knowledge of typhoid does not differ between VFR and non-VFR travelers,^38^ and an analysis of children returning from international travel has suggested typhoid fever is responsible for only 1% of systemic febrile illness in travelers.^39^ Therefore, the low reported knowledge of typhoid may be a symptom of low practical risks for infection. Among cases of travel-related hepatitis A, VFR travel has been identified in 52–64% of cases, with children accounting for the majority.^40,41^ Since 2006, hepatitis A vaccination has been routine in the United States^42^ but not in Canada, Australia, the United Kingdom, and other Western countries.

The definition of “VFR travelers” and “VFR travel” has been somewhat dynamic as interest in studying this group has increased.^43,44^ As such, the line between VFR travel as a behavior and VFR travelers as a cohort has not been clearly defined. Large, broad studies like those from GeoSentinal, which collect information on purpose of travel but do not expressly concentrate on VFR travel, are limited in their ability to draw conclusions about VFR travelers as a cohort, and therefore necessarily focus on VFR travel as a subset of travel behaviors.^17,18,39^ Because every member of our large study population has recently participated in VFR travel, and we collected data on non-VFR trips, our study presents a unique opportunity to assess this group at the cohort level.

Our analysis has several limitations. The survey was conducted online, and participants had to opt in. Thus, it is not clear how representative our sample is for the overall population of VFR travelers from the United States. Data were not captured regarding the specific regions visited within a country, which may have limited the accuracy of risk estimates. For example, risk for malaria is different in Bangkok from that in rural Thailand. Combining several countries into a single region for analysis may have similarly affected accuracy of risk estimates. For analysis purposes, expanding the definition of a VFR returning home traveler to include regions near the traveler’s home country and/or countries with similar risk profiles would have been preferred, but this was impractical given the data.

In future analyses, it may be advisable to adjust for a yellow fever vaccine requirement, which necessitates at least one visit to a travel physician. However, given that the yellow fever vaccine provides coverage for life, travel to a country that requires a yellow fever vaccine would not necessarily mean a person sought pretravel health care before an individual trip.

Given that certain demographic groups and travel variables are associated with both decreased odds of seeking pretravel care and increased odds of reporting post-travel illness, understanding these associations can help travel medicine doctors determine which patients are at greatest risk and how best to advise them about their travel plans. Data about knowledge and perceptions can also inform where more information is needed. Our results suggest greater education is needed regarding transmission and risk factors for hepatitis A, tuberculosis, typhoid, and Japanese encephalitis.

Findings from this study and others like it could serve as a means of helping travel medicine professionals focus on the most important issues during time-limited visits with patients. General practitioners could play an important role in educating first- and second-generation immigrants and referring them to local travel clinics, if possible. It is important for patients and doctors to have at least a general understanding of VFR risk factors and how they may differ depending on the purpose of travel. Discussion of vaccination is a particular priority for VFR travelers, who are more likely to decline recommended vaccines relative to other international travelers.^6,8^ Traveler-targeted interventions should encourage pretravel healthcare seeking to those who are least likely to seek it or have the greatest risk for disease. For example, programs could be developed that give uninsured or underinsured people opportunities to receive pretravel care. Online information—either general or more targeted, such as Facebook advertising—could be used to encourage travelers to visit travel clinics. It may also be possible to work with airlines to provide messaging during ticket purchases. Any educational initiatives should include outreach to and through community leaders.

Despite widespread evidence suggesting that VFRs are at increased risk while traveling, little progress has been made to reduce incidence of disease or increase participation in pretravel care seeking in the last several decades.^11^ There is little empirical evidence available to support the efficacy of community outreach and preventative health programs that have been implemented. Cultural and language barriers present a significant obstacle to reaching these communities, and culturally sensitive health promotion materials and training are lacking in travel medicine. These barriers, as well as potential concerns about cost, may also reduce the effectiveness of referral from a general practitioner to a travel clinic. Strengthening the evidence on which programs work and which do not is imperative, especially as the pool of data on VFR travelers and their risk factors grows.

Our analysis indicates low levels of pretravel health-seeking behaviors among first- and second-generation citizens and immigrants traveling internationally to at-risk countries, especially to countries of origin. In addition, VFR travelers commonly have gaps in understanding hepatitis A and typhoid transmission patterns. Future studies, including prospective studies, are needed to determine specific diseases and outcomes in VFR travelers to various regions, with an emphasis on VFR travelers as a cohort rather than a subset of travel behavior.

## Supplemental appendix

Supplemental materials
