# Supplementary material for: Surveying Health-Related Knowledge, Attitudes, and Behaviors of U.S.-Based Residents Traveling Internationally to Visit Friends and Relatives
Source: Am J Trop Med Hyg. 2020 Sep 21;103(6):2591–9. doi: 10.4269/ajtmh.20-0508 (PMC7695092; doi:10.4269/ajtmh.20-0508)
Supplement: Supplementary file 1 [file tpmd200508.SD1.pdf]

Supplemental Appendix A. Survey Text: Strategic Evaluation of VFR Segment for Travel Vaccines

Screening Questions:

1. Please confirm whether you are a full-time resident of the US.
   a. Yes
   b. No (terminate)
2. Please indicate your age group:
   a. Younger than 18 (terminate)
   b. 18-25 (terminate)
   c. 26-50
   d. 51-65
   e. 65+
3. Given that more than 40 million US residents were born outside the US, we're interested in where your family is from. In the box, please list the country where you/your mother/your father were/was born.
   a. Respondent (use drop down list of countries, USA should be the default)
   b. Father (use drop down list of countries, USA should be the default)
   c. Mother (use drop down list of countries, USA should be the default)

   Programmer note: QUOTA 75% FOREIGN BORN RESPONDENTS – respondent are parents not born in the US (25% 1ST GENERATION – respondent born in the US but parents not born in the US)
4. How many international trips have you taken in the past three years?
   a. 0 (terminate)
   b. 1
   c. 2
   d. 3-5
   e. 6-10
   f. More than 10
5. Of those trips, please indicate the reason for those international trips. (check all that apply)
   a. Vacation / Tourism
   b. Work / business trip
   c. Travelled to visit family or friends
   d. Education or school related trip
   e. Volunteer / international aid trip
   f. Other

   Program note: only participants selecting option "C" should advance, all others terminate
6. Including **ONLY** those trips that involved "**Visiting Family and Friends**":

(if 1 trip) please identify the primary country visited during your most recent international trip to visit family and friends.
(if 2 trips) (1st trip) please identify the primary country visited during the first of your two most recent international trips to visit family and friends.
(2nd trip) please identify the primary country visited during the second of your two most recent international trips to visit family and friends.
(if 3 trips) (1st trip) please identify the primary country visited during the first of your three most recent trips international trips to visit family and friends.
(2nd trip) please identify the primary country visited during the second of your three most recent trips international trips to visit family and friends.
(3rd trip) please identify the primary country visited during the third of your three most recent trips international trips to visit family and friends.

a. Trip #1 {DROP DOWN FROM COUNTRY LIST} (Dropdown Months) (Dropdown 2014-2017)
b. Trip #2 {DROP DOWN FROM COUNTRY LIST} (Dropdown Months) (Dropdown 2014-2017)
c. Trip #3 {DROP DOWN FROM COUNTRY LIST} (Dropdown Months) (Dropdown 2014-2017)
d. I have not visited family or friends outside the US in the past three years {IF SELECTED, STOP SURVEY}

Please indicate the month and year.

Program a terminate for Q6 if survey participants haven’t visited friends/family in at least one of the “at risk” countries listed at the top of the word document. Then only test for that specific country. A survey about someone’s trips to Europe to visit friends/family doesn’t help us very much but a trip to the “at risk” countries does.

Traveler Interview Questions
A. Recent International Travel Information

1. **We'd like to collect a little information about your 3 most recent international trips to visit family and friends:**
   For your September 2012 trip to (country from QS6),

**{1-6 IN A SINGLE CHART}**	**Trip 1**	**Trip 2**	**Trip 3**
1. How many TOTAL countries did you visit during this trip?			
2. Trip Length (days)	{DROP DOWN BETWEEN 0-100 days}	{DROP DOWN BETWEEN 0-100 days}	{DROP DOWN BETWEEN 0-100 days}
3. Who traveled with you from the US? Select all that apply<br>a. Spouse<br>b. Children<br>c. Other Family<br>d. Non-related adults<br>4. I traveled alone	{allow multiple answers}	{allow multiple answers}	{allow multiple answers}
5. Which of the following were the specific reasons for the trip? Select all that apply.<br>a. Visit Family<br>b. Visit Friends<br>c. Tourist Activities<br>d. Work Activities<br>e. Missionary / Aid work<br>e. Education / School Related	{allow multiple answers} - Still is a screening validation	{allow multiple answers} - Still is a screening validation	{allow multiple answers} - Still is a screening validation
f.			
**{7-13 IN A SINGLE CHART if**	**Trip 1**	**Trip 2**	**Trip 3**

**possible}**			
How many months in advance did you start planning the trip?	• 0-2 months<br>2 months or more	• 0-2 months<br>2 months or more	• 0-2 months<br>2 months or more
6. How many months in advance did you book air travel for the trip?	• 0-2 months<br>• 2 months or more	• 0-2 months<br>• 2 months or more	• 0-2 months<br>• 2 months or more
7. How did you make your travel arrangements? Select all that apply<br>a. Directly with airline<br>b. Directly with hotel<br>c. On-line travel service (orbitz, kayak, expedia, travelocity, etc)<br>d. Travel agent (US)<br>e. Travel agent (ex-US)<br>8. Other (explain in comment box)	• {allow multiple answers}	• {allow multiple answers}	• {allow multiple answers}
9. Prior to leaving for your trip which of the following did you do while planning for the trip? Select all that apply<br>a. Investigate visa requirements<br>b. Seek out travel health information<br>c. Research destination tourist activities<br>d. Buy gifts or US goods for family & friends<br>e. Traveled with OTC (Over the Counter) and other medications (e.g. diarrhea, upset stomach,	{allow multiple answers}	{allow multiple answers}	{allow multiple answers}

antibiotics, pain relievers, allergy) from US<br>f. None of the above			
f. Broadly please assess your health concerns about traveling on this trip?	1 (low risk) to 5 (high risk)	1 (low risk) to 5 (high risk)	1 (low risk) to 5 (high risk)
10. Where did you stay (Select all that apply)?<br>a. Hotels and/or Resorts<br>b. Home of family/friends<br>c. My home that I own<br>d. Short-term rental property (e.g. Airbnb property)<br>11. Other (please specify)	{allow multiple answers}	{allow multiple answers}	{allow multiple answers}
12. Please describe the areas where you stayed (Select all that apply):<br>a. Rural<br>b. Urban inner-city<br>c. Urban / Suburban<br>e. Other (explain in comment box)	{allow multiple answers}	{allow multiple answers}	{allow multiple answers}
d. {BELOW QUESTIONS TO BE ASKED SEQUENTIALLY FOR EACH TRIP. IF ONLY ONE TRIP WAS COMPLETED IN THE PAST THREE YEARS, ONLY ASK ONE SET OF QEUSTIONS. IF MULTIPLE TRIPS, ASK EACH SET OF QUESTIONS FOR EACH TRIP IN ORDER.			
a. Programmer note: If	{allow multiple	{allow multiple	{allow multiple

respondent selected B in question 10, continue sequentially. If B was <u>not</u> selected, please skip to question 21. You mentioned seeking travel health information prior to your (Month, Year) trip to (Location), which of the following sources did you use? Select all that apply Internet research<br>b. Spoke to or visited my health care practitioner (e.g. primary care practitioner, travel health specialist/clinic, pediatrician, pharmacy, government health clinic)<br>c. Asked family, friends and/or work colleagues<br>Other (explain in comment box)	answers}	answers}	answers}
14a. Programmer Note: Only ask if C (now B after editing answer choices) was selected for question 14.<br>Since you sought travel health information from your healthcare practitioner prior to your (Month, Year) trip to (Location), which of the following did you see? Select all that apply:<br>a. Primary care			

practitioner<br>b. Travel health specialist / clinic<br>c. Pediatrician<br>d. Pharmacy<br>d. Government health clinic			
13. For each member of your travel group, how many vaccines were recommended for your (Month, Year) trip to (Location)? *Programming note: only ask for those identified as traveling in question #5*<br>a. You (if checked, see program note)<br>b. Spouse (if checked, see program note)<br>c. Children (if checked, see program note)<br>d. Other Traveler (ask only if Q5=3/4)<br>PN: Skip Q16 and Q17 if Q13=Vaccinated from previous trip.<br>e. PN: Skip Q14-17 if None to all in Q13	Drop-down answers:<br>- None<br>- 1<br>- 2<br>- 3<br>- More than 3<br>- Vaccinated from previous trip<br>- Don't remember<br>PN: Skip Q16 and Q17 if Q13=None or Don't remember	Drop-down answers:<br>- None<br>- 1<br>- 2<br>- 3<br>- More than 3<br>- Vaccinated from previous trip<br>- Don't remember<br>PN: Skip Q16 and Q17 if Q13=None or Don't remember	Drop-down answers:<br>- None<br>- 1<br>- 2<br>- 3<br>- More than 3<br>- Vaccinated from previous trip<br>- Don't remember<br>PN: Skip Q16 and Q17 if Q13=None or Don't remember
14. For those receiving travel vaccine recommendations for your (Month, Year) trip to (Location), which of the following sources gave those recommendations? Select all	{allow multiple answers}	{allow multiple answers}	{allow multiple answers}

that apply<br>*Programming note: only ask to respondents receiving 1 or more vaccines recommendations in question #13 – no need to ask for each traveler*<br>a. Internet research<br>b. Healthcare Practitioner (e.g. primary care practitioner, travel health specialist / clinic, pediatrician, pharmacy, government health clinic)<br>c. Family, friends and/or work colleagues<br>Other (explain in comment box)			
16a. Programmer Note: Only ask if C (now B after editing answer choices) was selected for question 15 (previously question 14).<br>Since you received travel vaccine information from your healthcare practitioner for your (Month, Year) trip to (Location), which of the following gave you that information? Select all that apply:<br>a. Primary care practitioner<br>b. Travel health			

specialist / clinic<br>c. Pediatrician<br>d. Pharmacy<br>d. Government health clinic			
15. For those travelers who were recommended travel vaccines for your (Month, Year) trip to (Location), please indicate if each person received the vaccines:<br>a. All (ask #16)<br>b. Some (ask open end – how did you determine which to accept and which not) (Ask Q18 and Q19)<br>e. None (ask #17) (Skip Q18)			
c. Programmer Note: For all travelers that were recommended vaccines (checked in question #13), please ask questions #16 or #17 individually for each traveler, I would call them 17b,17c,17d and 16d,16c,16d)			
16. If yes, which of the following reasons best explains why you accepted vaccines for your (Month, Year) trip to (Location)?<br>a. Peace of mind<br>b. To protect against disease<br>c. Travel visa requirements<br>d. Appease family member	{DROP DOWN LIST WITH ANSWERS}	{DROP DOWN LIST WITH ANSWERS}	

Doctor recommendation			
17. If not, which of the following reasons best explains why you refused vaccines?<br>a. Concerned about how safe the vaccine is<br>b. Concerned about how well the vaccine works<br>c. Concerned about the cost of the vaccine<br>d. Not concerned with illnesses<br>e. Fear of needles / injection<br>f. Did not know enough about the disease the vaccine was protecting against<br>e. Timing, logistic, or access challenges	{DROP DOWN LIST WITH ANSWERS}	{DROP DOWN LIST WITH ANSWERS}	{DROP DOWN LIST WITH ANSWERS}
18. How much out of pocket did you spend on vaccinations per person for your (Month, Year) trip to (Location)?<br>a. $0<br>b. $1-$49<br>c. $50-$99<br>d. $100-$249<br>e. $250-$500<br>g. More than $500			
19. Of those traveling for your (Month, Year) trip to (Location), please indicate if anyone got sick while traveling or shortly after returning to the US? Select all that apply<br>a. You			

b. Spouse<br>c. Child<br>d. Other traveler<br>a. None of the above			
20. Please explain what actions were required to treat the sick person either locally in (Location) or back in the US)? Select all that apply<br>*Programming note: only ask to respondents who had a sick traveler identified in question #19*<br>a. Local Physician visit<br>b. Local Hospital visit<br>c. Local Hospital overnight<br>d. Prescription only medication required<br>e. Over-the-Counter medication required<br>f. Treatment in the US<br>e. None of the above			
21. Which of the following food related practices did you follow while on your (Month, Year) trip to (Location)? Please select all that apply<br>a. Drank tap water in local homes<br>b. Drank tap water in hotels/resorts<br>c. Brushed teeth with tap water<br>d. Consumed fruits and vegetables purchased at			

local supermarket<br>e. Consumed meats purchased at local supermarket<br>f. Consumed locally prepared street food<br>g. None of the above			
22. On a scale of 1-5, how would you rate your adherence to safe food, water, and hygiene practices during your (Month, Year) trip to (Location) with 1 being poor and 5 being best?	{DROP DOWN LIST WITH NUMBERS}	{DROP DOWN LIST WITH NUMBERS}	{DROP DOWN LIST WITH NUMBERS}

B. Travel Related Health Risks

27. Which of the following websites did you use while researching health information for any of your recent trips? *Programming Note: only ask respondents that did health info search in Question #9*
    a. www.TravelVaccine.com
    b. www.PasportHealthUSA.com
    c. www.IAMAT.com
    d. www.CDC.gov
    e. www.WHO.org
    f. Other (Explain in comment box)
    g. Not aware of any of the listed websites
28. Not including the trips discussed previously, have you ever been vaccinated prior to international trips in the last three years?
    a. Yes (please specify which countries you visited that required vaccination)
    b. No (skip #29)
29. Who suggested getting vaccinated for your previous international travels?
    a. Primary Care Physician
    b. Travel Health Specialist
    c. Friends or work colleague
    d. Family
    e. Employer
    f. I did my own research
30. Please indicate how familiar you are with the following diseases.
31. Please indicate how you believe each disease is contracted.
32. Please indicate your personal risk of contracting each disease in the area(s) you stayed in during your (Month, Year) trip to (Location) countries from S6)
33. Please indicate the relative risk of infection for any traveler in the area(s) you stayed in during your (Month, Year) trip to (Location) (show countries from S6)

Cholera	5 – Very Aware<br><br>4 – Somewhat Aware<br><br>3 – Not Very Aware<br><br>2 – Recognize disease, but not familiar<br><br>1 – Not at all familiar with this disease	a. Contaminated food and/or water<br>b. Contact with contaminated bloodily fluids (e.g. blood, saliva)<br>c. Airborne transmission<br>d. Insect bite<br>e. Animal bite	5-Very high risk<br><br>4-Somewhat high risk<br><br>3-Moderate risk<br><br>2-Somewhat low risk<br><br>1-Very low risk	5-Very high risk<br><br>4-Somewhat high risk<br><br>3-Moderate risk<br><br>2-Somewhat low risk<br><br>1-Very low risk
Hepatitis B	Same as above	Same as above	Same as above	Same as above
Hepatitis A				
Malaria				

Tuberculosis				
Typhoid				
Rabies				
Japanese Encephalitis				

C. Attitude statements:
How much do you agree or disagree with the following statements?

	Strongly Agree	Agree	Neutral	Disagree	Strongly Disagree	Don't know / Not Sure
34. Childhood vaccines are safe, effective and necessary for the well being of a healthy population						
35. Appropriate and targeted travel vaccine use is safe, effective and necessary for the well being of a healthy population						
36. Travel vaccines are as safe and effective as childhood vaccines						
37. I am concerned of a serious negative reaction to receiving travel vaccines?						

D. General Demographic Information

38. What is your marital status?
    a. Married
    b. Single
    c. Divorced
    d. Other
39. In addition to you, how many additional people live in your household?
    a. How many children (defined as 18 years old or less)?
    b. How many adults (defined as 19-59 years old)?
    c. How many elderly (defined as over 60 years old)?
40. Do you own your home or do you rent?
    a. Own
    b. Rent
41. What educational milestones have you achieved and where did you study?

	US-based School	School Outside of US
a. High school diploma		
b. Trade school diploma		

c. College degree		
d. Master's degree		
e. Doctorate degree		

42. Considering languages that you speak (select up to 3), please indicate:
    a. Your level of fluency
    b. If the language is frequently spoken in your home

**Languages Spoken**	**a. Level of Fluency**		**b. Percent of Time Language is Spoken at Home**
	Conversational Fluency	Native-level Fluency	
1. {DROP DOWN LIST OF Languages}<br>Other specify			0%<br>25%<br>50%<br>75%<br>100%
2. {DROP DOWN LIST OF Languages}<br>Other specify			0%<br>25%<br>50%<br>75%<br>100%
3. {DROP DOWN LIST OF Languages}<br>Other specify			0%<br>25%<br>50%<br>75%<br>100%

43. Which nationalities do you identify with most strongly? Select up to three:
    a. {DROP DOWN LIST OF COUNTRIES FROM ABOVE; USA should be first on list}
    b. {DROP DOWN LIST OF COUNTRIES FROM ABOVE; USA should be first on list}
    c. {DROP DOWN LIST OF COUNTRIES FROM ABOVE; USA should be first on list}
44. Given that you live in the US, what are the primary ways that you stay connected to your home country culture. Select all that apply:

a. Live within an immigrant community
b. Support local businesses owned by other immigrants
c. Member of local social club / organization with other immigrants
d. Websites/Newspaper/Magazines from home country (Please specify: ____)
e. Attend religious services with other immigrant families
f. Watch or attend sporting events from home country
g. Watch television stations or movies from home country (Please specify: ____)
i. Facebook Other Social Media Telephone calls to family and friends in home country
j. Other (Explain in comment box)

45. Which of the following best describes the industry you work in? {AUTOFILL DROPDOWN}
    a. Agriculture (including forestry, fishing, and hunting)
    b. Manufacturing
    c. Construction
    d. Mining
    e. Utilities
    f. Wholesale trade
    g. Retail Trade
    h. Transportation and warehousing
    i. Information
    j. Financial activities
    k. Professional and business services
    l. Educational services
    m. Health care and/or Social Services
    n. Leisure and hospitality
    o. Government/public administration
46. What is your job title?
    p. {COMMENT BOX}
47. Do you travel internationally for your job?
    a. Yes
    b. No
48. How many times in the past 12 months did you visit a doctor or otherwise receive medical care?
    a. 0
    b. 1x-2x
    c. 3x-5x
    d. 5x+
49. Where do you typically receive your medical care? Select all that apply
    a. Physician office
    b. Local hospital
    c. Community medical center
    d. Retail walk-in or urgent care medical clinic (e.g. CVS Minute Clinic)
    e. Emergency room at hospital
    f. Other (Explain in comment box)
50. How long have you been seeing your regular Primary Care Physician?
    a. Don’t have a regular Primary Care Physician
    b. Less than one year
    c. 1-5 years
    d. 5-10 years
    e. 10+ years
51. Do your children have a regular pediatrician that is different than your Primary Care Physician?
    a. Yes

b. No
c. Does not apply (children are too old for pediatrician or do not have children)

52. Do you think any of your regular healthcare providers have practices that have a large cohort of recent immigrant patients or might even be immigrants themselves?
    a. Yes
    b. No
    c. Not sure
53. What type of medical insurance do you have?
    a. Commercial through employer
    b. Commercial not through employer
    c. Medicare or other government health coverage
    d. None
