# Supplementary material for: Surveying Health-Related Knowledge, Attitudes, and Behaviors of U.S.-Based Residents Traveling Internationally to Visit Friends and Relatives
Source: Am J Trop Med Hyg. 2020 Sep 21;103(6):2591–9. doi: 10.4269/ajtmh.20-0508 (PMC7695092; doi:10.4269/ajtmh.20-0508)
Supplement: Supplementary file 2 [file tpmd200508.SD2.pdf]

Supplemental Appendix B. Survey Responses

	N (%)
**Started survey (excluding Mexican descent)**	**8961 (100)**
Demographic exclusions	
Not full time US resident	426 (5)
Under 26	774 (9)
At least one U.S.-born parent	5060 (56)
**Potential adult VFR travelers**	**2701 (30)**
Travel exclusions	
No travel in last 3 years	519 (6)
No VFR travel	779 (9)
No “at-risk” travel*	319 (4)
Survey failures	90 (1)
**Total completed survey**	**994 (11)**

VFR, visiting friends and relatives.
*At-risk travel was defined as visiting a country with a typhoid vaccine recommendation
